# Surveillance and Treatment of Non-Muscle-Invasive Bladder Cancer in the USA

**DOI:** 10.1155/2012/421709

**Published:** 2012-05-10

**Authors:** Daniel A. Barocas, Denise R. Globe, Danielle C. Colayco, Ahunna Onyenwenyi, Amanda S. Bruno, Thomas J. Bramley, Rachel J. Spear

**Affiliations:** ^1^Department of Urologic Surgery, Vanderbilt University, Nashville, TN 37203, USA; ^2^Allergan Inc., P.O. Box 19534, Irvine, CA 92623, USA; ^3^Xcenda, 4114 Woodlands Parkway Suite 500, Palm Harbor, FL 34685, USA

## Abstract

Seventy percent of newly diagnosed bladder cancers are classified as non-muscle-invasive bladder cancer (NMIBC) and are often associated with high rates of recurrence that require lifelong surveillance. Currently available treatment options for NMIBC are associated with toxicities that limit their use, and actual practice patterns vary depending upon physician and patient characteristics. In addition, bladder cancer has a high economic and humanistic burden in the United States (US) population and has been cited as one of the most costly cancers to treat. An unmet need exists for new treatment options associated with fewer complications, better patient compliance, and decreased healthcare costs. Increased prevention of recurrence through greater adherence to evidence-based guidelines and the development of novel therapies could therefore result in substantial savings to the healthcare system.

## 1. Introduction

Non-muscle-invasive bladder cancer (NMIBC), formerly known as superficial bladder cancer, is a common, heterogeneous disease associated with high rates of recurrence and that often requires lifelong surveillance [1–4]. Treatment options for NMIBC are limited, with initial management involving transurethral resection of the bladder tumor (TURBT), followed by adjuvant instillations of chemotherapy or immunotherapy to reduce recurrence rates and prevent disease progression [3]. Commonly used current intravesical therapies include mitomycin C—a naturally occurring product of Streptomyces bacteria that has antibacterial and antitumor properties—and bacillus Calmette-Guérin (BCG), an attenuated mycobacterium that produces an inflammatory reaction in the bladder. Both of these therapies were introduced several decades ago and have been shown to reduce recurrence rates. Each of the intravesical therapy options has associated toxicities that can impair patient compliance, particularly so for BCG, which routinely causes irritative voiding symptoms, often causes fever and malaise, and in rare cases, results in systemic sepsis [5–9].

Clinical guidelines for NMIBC aim to guide clinicians to appropriate use of intravesical therapy; however, guidelines from the different associations vary in their recommendations, so the physician's subjective assessment of the benefits and the patient's preferences (such as acceptability of risk) can supersede the available evidence favoring the use of intravesical therapy [10–16]. As a result, real-world utilization of intravesical therapy often falls below the recommendations, resulting in potentially preventable bladder cancer recurrences and associated downstream consequences, such as disease progression and reduced quality of life [17, 18]. Cystectomy is typically reserved for patients with high-risk NMIBC who elect for early, aggressive surgical intervention, for those who have failed intravesical therapy, and for those who have progressed to muscle-invasive disease [10, 12, 14, 19–24].

In addition to the clinical burden of bladder cancer, the disease is associated with a high economic burden, accounting for the highest lifetime treatment costs per patient of all cancers due to its high recurrence rate, long-term survival rate, and costs associated with disease surveillance and treatment [25, 26]. Potentially preventable recurrences due to underuse of intravesical therapy may add substantially to the cost of caring for patients with NMIBC [27].

There are many unmet needs in the treatment of NMIBC, which contribute to its burden on both patients and society. Thus, the objective of this paper is to qualitatively review the societal and economic burden associated with the disease in the USA and to describe the shortcomings of currently available NMIBC treatments. This paper will underscore the need for new treatment options that are associated with fewer complications, better patient compliance, increased utilization in appropriate settings, and lower healthcare costs.

## 2. Methods

This qualitative review was conducted by applying a list of search terms to a database of the medical sciences (PubMed, published by the National Library of Medicine). The set of search terms included variations of “bladder cancer,” “NMIBC,” “BCG,” “mitomycin,” “intravesical therapy,” “cystectomy,” “epidemiology,” “risk factors,” “guidelines,” “efficacy,” “clinical burden,” “toxicity,” “diagnostic,” “health-related quality of life,” and “economic burden.” Articles were excluded if they were published in languages other than English. A total of 98 articles were identified, abstracted, and evaluated using the Oxford Center for Evidence-Based Medicine's 2011 levels of evidence [28]. The majority of those were then used in this paper to describe current treatment options, the clinical and economic burden of NMIBC, and unmet needs of NMIBC that exist today.

## 3. Overview of NMIBC

The bladder is a vessel that stores urine produced by the kidneys before excretion [4]. The bladder itself is made up of several tissue layers. The first 2 layers include the urothelial and the lamina propria layers. The urothelial or mucosal layer makes contact with the bladder contents, while the lamina propria, or submucosal layer, connects the urothelial layer to the underlying smooth muscle [4]. Bladder cancer is a common malignancy arising from the urothelial cells and is responsible for considerable morbidity and mortality [2]. The most common symptom of bladder cancer is hematuria, which occurs in 80% to 90% of patients [4]. Approximately 70% of newly diagnosed cases of bladder cancer are NMIBC, meaning that they are confined to the urothelial and lamina propria layers of the bladder [3, 4, 26, 29–32]. Among NMIBCs, around 70% present as Ta lesions (papillary tumor confined to the urothelium), 20% as T1 lesions (tumor invades the lamina propria), and 10% as carcinoma *in situ* (CIS) (flat, high-grade tumor confined to the urothelial layer) [4, 29].

NMIBC tends to require lifelong surveillance, due primarily to the fact that disease recurrence occurs frequently, even in those patients treated with TURBT and existing intravesical therapies [3, 6–8, 13, 14, 24, 32–45]. TURBT is the standard initial treatment option for NMIBC, which can be performed with or without intravesical chemotherapy, as a one-time immediate postoperative instillation [35, 46]. The most commonly used medication for perioperative instillation is mitomycin C. TURBT is both diagnostic and therapeutic, and the procedure provides critical staging information [35]. TURBT may be followed by an induction course (with or without subsequent maintenance courses) of intravesical therapy, given as a series of treatments over several weeks [35, 46]. The current intravesical treatment options, such as BCG and mitomycin C, are associated with side effects, complications, and a lack a consistent role in the therapy of NMIBC and often go underutilized.

## 4. Epidemiology and Risk Factors

Bladder cancer is one of the most commonly diagnosed malignancies in the USA, with an estimated 70,530 new cases diagnosed in 2010 [1]. Its effect on mortality is also substantial, with a projected 14,680 deaths that same year [1]. However, in terms of stage distribution, bladder cancer has a 5-year relative survival rate—which measures the survival of the cancer patients in comparison to the general population—of 97% for *in situ*, 71% for local, 35% for regional, and 5% for distant cases [47].

Bladder cancer commonly affects the elderly, with the median age of 73 years at diagnosis [47]. Bladder cancer incidence varies in terms of gender and ethnicity. The lifetime risk of developing bladder cancer is 3.81% for males and 1.18% for females, and the risk escalates with increasing age [1]. Bladder cancer is the fourth-most-common cancer in men in terms of new cases, with an incidence 4 times higher than women (37.9 versus 9.6 per 100,000) [1]. In terms of ethnicity, the incidence of bladder cancer is approximately 2 times higher in white men than in African American men in the USA, although African Americans tend to present at a higher stage and with a lower median survival [1, 48].

Risk factors for bladder cancer include cigarette smoking; work-related contact with cyclic chemicals (e.g., benzene and arylamines); and exposure to dyes, rubbers, textiles, and paints [4, 49, 50]. Cigarette smoking is the number-one environmental risk factor, attributable to 50% to 66% of bladder tumors in men and 25% in women [4]. Genetic factors also appear to influence the risk of developing bladder cancer, possibly through metabolism of environmental carcinogens [49].

## 5. Clinical Outcomes of NMIBC

Important clinical outcomes in the natural history of NMIBC include recurrence and progression. Patients with NMIBC have a high risk for disease recurrence (i.e., tumors of the same stage and grade as primary tumor), or disease progression (i.e., a higher stage with muscle invasion or metastasis throughout the clinical course of treatment), even in those treated with TURBT and existing intravesical therapies [3, 6–8, 13, 14, 24, 32–45]. Nearly one-third of patients with NMIBC will have a recurrence of disease within 2 years, and patients often require routine monitoring and treatment for the rest of their lives [2, 15, 26, 31, 46, 51–56].

There are several different ways to categorize a patient's risk of recurrence. For example, the European Organization for Research and Treatment of Cancer (EORTC) has provided risk tables, which allow calculation of an NMIBC patient's probability of recurrence and progression after TURBT [14]. The calculation is based on 3 clinical factors (number of tumors, tumor size, prior recurrence rate) and 3 pathological factors (T category, CIS, tumor grade) [20]. The total score is then calculated, and each patient falls into a low-, intermediate-, and high-risk group for the recurrence and progression risk groups [14]. It is important to note that the 5-year risk of progression to advanced disease and recurrence has been reported to range from 1% to 45% and 31% to 78%, respectively, in patients with NMIBC [51]. Even though adjuvant intravesical chemotherapy does not remove all the risk of recurrence, it is still essential to consider it in all patients to decrease the risk of recurrence [14].

In addition to recurrence and progression, complications of disease surveillance and treatments can contribute to the clinical burden. Disturbances in a patient's urinary habits are often noticed (including urinary tract complications such as infection, inflammation, urinary incontinence, urinary obstruction, renal insufficiency, and bladder perforation) [2].

## 6. Treatment of NMIBC

Considerations for appropriate treatment options in non-muscle-invasive disease must be made in conjunction with clearly defined goals—namely, to prevent disease recurrence and progression to muscle-invasive disease and to avoid the loss of the bladder [4]. The standard initial treatment supported for NMIBC is TURBT, which can be performed on an outpatient basis with general anesthesia [13, 14, 16, 35, 57]. The most common side effects associated with TURBT are bleeding and infection [4]. To prevent tumor recurrence, TURBT may be followed by an immediate postoperative instillation of chemotherapy (e.g., mitomycin C, epirubicin, and doxorubicin) and/or an adjuvant course of intravesical chemotherapy or immunotherapy (e.g., BCG). The typical adjuvant course consists of a 6-week induction (to induce remission) of intravesical chemotherapy given via a urethral catheter and left in the bladder for a specific amount of time, usually 1 to 2 hours [4, 7, 13]. In addition, a patient may go on to receive a maintenance regimen, typically consisting of 3 weekly instillations at 3, 6, 12, 18, 24, 30, and 36 months from start of induction [8].

The antibiotic chemotherapeutic agent mitomycin C is considered a standard of care for bladder instillations after TURBT [3]. One study of patients with low-risk NMIBC (stage Ta and T1 disease) found that single-dose mitomycin C resulted in a significantly lower early recurrence rate (11% versus 21% for placebo) within the first 24 months following initial TURBT [58]. Another study was performed with 502 patients (stage Ta or T1 disease) randomized after TURBT to either no further treatment, 1 instillation of mitomycin C immediately postoperatively, or 1 instillation of mitomycin C immediately postoperatively plus intravesical instillations at 3-month intervals for a year [59]. After a median follow-up of 7 years, a single instillation of mitomycin C decreased tumor recurrence by 50% compared with those patients who did not receive any intravesical therapy [59]. Thus, in patients with NMIBC at low risk of recurrence and progression, 1 immediate instillation of chemotherapy is recommended after TURBT [14]. Use of immediate instillation of chemotherapy after TURBT is discussed in more detail here in after under the section Treatment Guidelines for NMIBC.

Mitomycin C is commonly administered at dosages of 20 to 60 mg weekly for 6 to 8 weeks and in some instances followed by maintenance therapy monthly for a year [4]. Even though some literature has suggested that maintenance therapy enhances the effectiveness of mitomycin C induction in preventing tumor recurrence, its role is still uncertain [13, 16]. For example, according to Tolley et al., there was not conclusive evidence that 5 instillations of mitomycin C for a year offered significant benefit over the single instillation [59]. According to a meta-analysis involving mitomycin C, thiotepa, and epirubicin, a short intensive schedule of instillations within the first 3 to 4 months after an immediate instillation may be as effective as longer-term treatment schedules [60]. Also, higher drug concentrations and providing drug concentration optimization in the bladder have been suggested to potentially provide better results [60]. Thus, data examining duration and frequency of instillations of intravesical chemotherapy for patients whose disease has recurred are generally inconsistent and do not support use of any chemotherapy treatment longer than 1 year [14, 16].

Mitomycin C has a high molecular weight, resulting in a low incidence of systemic side effects [4]. In particular, the most common adverse effect of mitomycin C is chemical cystitis, which has been reported in up to 41% of patients [7]. The manifestations of cystitis include dysuria, frequency, urgency, suprapubic pain, and discomfort. In addition, the incidence of decreased bladder capacity has been as high as 22% in clinical trials, with the rare need for cystectomy due to severe bladder contractures [61]. Less common adverse effects include eczema-like reactions (4% to 12%) and myelosuppression, which is rare [5].

In addition to mitomycin C, 4 other immediate intravesical chemotherapy agents are available and used in the USA: doxorubicin, epirubicin, thiopeta, and valrubicin. Mitomycin C, epirubicin, and doxorubicin all have similar efficacy within the setting of immediate intravesical instillation to mitomycin C [45]. The anthracycline antibiotic-related chemotherapy agents include doxorubicin, epirubicin, and valrubicin. Their mechanism of action involves DNA inactivation and the production of activated oxygen radicals to interfere with cancer cell function [7]. Valrubicin is specifically indicated for use in patients with BCG-refractory CIS; however, it plays no role in the treatment of NMIBC in Europe [13]. Epirubicin and valrubicin may have a slight advantage with fewer side effects compared to doxorubicin but have similar side effects as mitomycin C [7]. Up to 50% of patients who have received doxorubicin have experienced symptoms of chemical cystitis [7]. Local toxicity seems to be lower with epirubicin and valrubicin and includes cystitis, dysuria, and increased urinary frequency and urgency [5]. Thiotepa is another option for immediate instillation but is rarely used in the USA because of its associated risk of myelosuppression [57].

New drug delivery systems, such as electromotive drug administration (EMDA), have been explored in order to enhance the effectiveness of intravesical chemotherapy [62]. In a randomized study comparing passive mitomycin C to EMDA mitomycin C for patients with high-risk NMIBC, time to recurrence was prolonged to 35 months with EMDA, compared with 19.5 months with passive mitomycin C (*P* = 0.013) [63]. Further study is warranted to assess the feasibility of implementing EMDA in the larger uro-oncologic community.

As an adjuvant agent, the intravesical immunotherapy agent BCG is commonly cited as the superior choice for high-risk patients due to the benefit/risk profile. In a meta-analysis that compared mitomycin C versus BCG in patients with intermediate- or high-risk NMIBC, those patients receiving BCG induction with maintenance saw a 32% reduction in the risk of recurrence compared to those receiving mitomycin C (*P* < 0.0001), while there was a 28% increase in the risk of recurrence (*P* = 0.006) for BCG in the trials without BCG maintenance [64]. In another meta-analysis, a 27% reduction in the odds of progression was demonstrated with the addition of BCG maintenance treatment for patients with NMIBC (*P* = 0.0001) [65]. The size of the reduction was similar in patients with Ta and T1 papillary tumors and in those with CIS [65]. Because of these data, the European Association of Urology (EAU) recommends that in those patients with tumors at high risk of progression, at least 1 year of maintenance therapy with BCG is indicated [14].

Even though maintenance therapy with BCG has been shown to be more effective than chemotherapy at preventing recurrences in patients with tumors at high risk of progression, the widespread use of BCG is limited due to its adverse effect profile [3, 7, 12–14, 20, 23, 30, 35, 41, 43, 66]. BCG is associated with complications spanning from mild fever, hematuria, hepatitis, cystitis, and tuberculosis to life-threatening sepsis [5–9, 31]. Most importantly, one-third of eligible patients starting BCG completed the full 3-year maintenance treatment schedule, with 20% stopping treatment because of BCG toxicity [67]. Since the major complications appear after systemic absorption, BCG should not be administered during the first 2 weeks after TURBT, when the risk of systemic absorption is highest [14]. Therefore, BCG has no role in the immediate postoperative setting [59].

When deciding on the optimal treatment option for NMIBC, there should be careful consideration of the patients' risk of recurrence and progression, along with the side effects of each treatment. While all of these chemotherapeutic instillations to some degree may be safe and effective at reducing recurrence risk in the short term, recurrences remain common in the long term. As a result, patients must be monitored at intervals, because these recurrences are associated with a substantial economic burden for the healthcare system, a decrement in the quality of life for patients, and, ultimately, leave the patients at risk for a poor cancer control outcome [3].

Radical cystectomy is the standard of care for BCG-refractory CIS and serves as an option for patients with NMIBC who have high-risk disease and desire aggressive intervention, who have recurred after intravesical therapy, or who have progressed to muscle-invasive disease; however, its use is associated with a 3% risk of mortality and a 28% to 60% risk of morbidity [10, 12, 22, 24, 31, 33, 36, 38, 68–70]. Furthermore, it is an invasive surgical procedure, associated with a high risk of infection and bleeding, a lengthy recovery period, and significant alterations in body image and urinary, sexual, and gastrointestinal function [4].

New agents that reduce the short-term risk of recurrence with improved safety and efficacy are needed for those with superficial bladder cancer. One of these promising agents in development for the management of NMIBC is apaziquone. Apaziquone, an indolequinone chemotherapeutic agent, has shown little systemic absorption or toxicity when used intravesically and had activity similar to mitomycin C, BCG, and epirubicin when tested against low-grade marker lesions. In a marker lesion study, the complete response rate after 6 consecutive instillations of apaziquone in patients with superficial bladder cancer was 67% (95% confidence interval [CI]: 51%, 80%) [71]. Local side effects were shown to be similar to those due to mitomycin C and epirubicin, but less severe and less frequent compared to BCG [71].

Other agents that are being investigated include gemcitabine and docetaxel. Gemcitabine has been shown to have high response rates, with complete response rates of 46.4% among those with superficial bladder cancer [72]. However, patients are not disease-free for an extended period of time, with 67.8% of patients experiencing recurrence during the first year [72]. In a phase 1 trial, docetaxel demonstrated a complete durable response in 22% of patients and a median disease-free survival of 13.3 months [73].

## 7. Treatment Guidelines for NMIBC

Current treatment guidelines support the use of a single dose of adjuvant intravesical chemotherapy for low-risk patients with NMIBC because of its ability to reduce the risk of tumor recurrence [14, 16]. A meta-analysis of 7 randomized clinical trials found a 39% decreased odds of recurrence at a median follow-up of 3.4 years in patients who received a single postoperative instillation of chemotherapy versus patients receiving TURBT alone [45]. In 2005, a review by an international consensus panel found that the risk of recurrence can be reduced by 50% at 2 years and by 15% or more at 5 years with a single dose of adjuvant intravesical chemotherapy [43]. Accordingly, the panel recommended a single dose of intravesical chemotherapy, ideally within 6 hours, and no more than 24 hours after TURBT, with the exception of patients with a suspected bladder perforation [43].

For patients with a small-volume, low-grade Ta bladder cancer, the American Urological Association (AUA) guidelines advance an initial single postoperative dose of intravesical chemotherapy as an option [16]. The EAU recommends 1 immediate postoperative instillation of chemotherapy to reduce the risk of recurrence in all eligible Ta and T1 tumors [14].

Despite the recommendation of immediate intravesical chemotherapy in current treatment guidelines, actual use is thought to be low, with estimates varying widely. A study of 14,677 bladder cancer patients undergoing TURBT between 1997 and 2004 found that only 49 (0.33%) received intravesical chemotherapy within 24 hours [15]. Whereas, according to data from the Surveillance, Epidemiology, and End Results (SEER) Program from 2004 to 2007, only 6.7% of patients diagnosed with Ta bladder cancer received an intravesical perioperative instillation of chemotherapy with or without subsequent induction therapy [74]. In the Bladder Cancer Patterns of Care project, 31.5% of NMIBC patients received adjuvant intravesical instillation [17].

The management of NMIBC is highly variable due to several factors, including divergence in treatment-related evidence. A retrospective database analysis examined compliance with established treatment guidelines during the initial 2 years after diagnosis as well as disease-specific survival after the initial 2-year period [18, 75]. Of the 4,545 individuals studied, only 1 received all the guideline-recommended care, despite level 1 evidence (intravesical therapy) [18]. Individuals compliant with the measures had a lower risk of mortality (hazard rati: 0.4; 95% CI: 0.18, 0.89) compared to those who received fewer than 4 cystoscopies, fewer than 4 cytologies, and no BCG [75]. This research confirms that there is generally poor adherence to evidence-based guidelines in the current NMIBC patient population, and that such adherence substantially improves outcomes.

## 8. Economics of Bladder Cancer

Overall, bladder cancer is the ninth-most costly cancer in the US and was estimated to cost approximately $3.98 billion in 2010 [76]. Assuming a 2% annual increase in direct medical costs in the initial and final phases of care, projected 2020 costs increase to $4.9 billion (in 2010 dollars) [76]. Furthermore, because these cost estimates do not include other types of costs, such as lost productivity or patient suffering, the total economic burden of bladder cancer is considerably higher. Based exclusively on direct medical costs, however, bladder cancer is the most costly of all cancers in terms of lifetime per-patient treatment costs, which have been estimated to range from $96,000 to $187,000 per patient in 2001 US dollars [25]. This high cost of bladder cancer is largely due to the disease's high recurrence rate and low mortality rate and costs associated with disease surveillance [25, 26].

Treatment of complications associated with bladder cancer therapy contributes to almost one-third of total costs [2]. Also, treating recurrences constitutes a significant portion of the costs of bladder cancer care, as a recent, retrospective review of 208 bladder cancer patients estimated that 60% ($39,393) of the lifetime cost per patient ($65,158) was attributable to surveillance and treatment of recurrences [2]. Thus, prevention of recurrences by adherence to evidence-based guidelines and development of novel therapies that decrease rate of recurrence and progression with a better side effect profile could result in substantial savings to the healthcare system, thereby increasing the importance of additional research and funding in this area [77].

## 9. Quality of Life

Treatment-associated complications not only contribute to the economic burden of bladder cancer but also increase patient impairment in physical health and worsened quality of life [78–80]. Quality of life affecting patient care and patient satisfaction with treatment options is becoming increasingly important by the medical community at large [80]. While there are several quality of life instruments in bladder cancer, no predominant single index is used widely [81].

Overall, bladder cancer patients report a statistically significant decline in physical health compared to control subjects and greater mean reductions in general well-being as measured by the SF-36 than patients with prostate and breast cancer [78]. In addition, general health perceptions (as measured by the SF-36) in NMIBC patients compared with population norms have been shown to be severely impaired [82]. With the implementation of the first few TURBT procedures, physical functioning, social functioning, and role-emotional domains have also been shown to demonstrate impairment [82].

Two studies that assessed the impact of intravesical instillation on quality of life found that BCG induction resulted in an acute, moderate deterioration in quality of life following instillation [83, 84]. Kulkarni et al. [85] estimated the disutilities associated with treatment of NMIBC, quantified in quality-adjusted life years (QALYs), and showed that treatment leads to significant decrements in the quality of life. Thus, efforts to reduce preventable recurrences should lead to substantial humanistic benefits [86].

## 10. Conclusion

Bladder cancer is a heterogeneous, highly prevalent disease that accounts for significant morbidity and mortality in the USA [2, 3]. The high rates of recurrence and risk of disease progression in bladder cancer often require lifelong surveillance, making the disease both clinically and economically important [51]. Further research is needed to quantify the humanistic burden of NMIBC using instruments validated in the appropriate patient populations.

Even with current therapies, recurrences are common, and the efficacy of treatments must be balanced with their toxicity so that no single treatment option can be considered superior across all NMIBC cancer risk strata and all individual patients. This creates an unmet need for new treatment options associated with fewer complications, better patient compliance, improved utilization in appropriate settings, and lower costs [3, 12, 13, 20, 41–43]. Perhaps the greatest opportunity lies in the use of perioperative intravesical therapy, as the large gap between the guideline recommendations and apparent utilization may be improved by physician adherence to guidelines.

Fortunately, new treatment options that can be used immediately after surgical removal of bladder tumors in patients with NMIBC are currently being tested and have been shown to increase the time to recurrence of disease and are also associated with less frequent and less severe adverse events compared to the most commonly used intravesical therapies [71]. Treatments such as apaziquone may have clinical and economic advantages for intravesical use in patients with NMIBC and may gain wider acceptance among treating physicians. Further research in the optimal treatment of NMIBC, from both a clinical and economic standpoint, is warranted.
